# Enhancing the Corrosion Resistance of AlCoCrFeNi High-Entropy Alloy Coatings via TiO_2_ Doping

**DOI:** 10.3390/molecules31183205

**Published:** 2026-09-11

**Authors:** Ying Wang, Yan Xiong, Shuobin Chen, Mao Zhang, Yuxuan Liu, Zhigang Hu, Ming Ma

**Affiliations:** 1 School of Mechanical Engineering, Wuhan Polytechnic University, Wuhan 430023, China; 2 College of Artificial Intelligence, Wuchang College of Technology, Wuhan 430200, China; 3 State Key Laboratory of Materials Processing and Die & Mould Technology, School of Materials Science and Engineering, Huazhong University of Science and Technology, 1037 Luoyu Road, Wuhan 430074, China

**Keywords:** high-entropy alloy coating, laser cladding, TiO_2_ doping, corrosion behavior

## Abstract

This study investigated the corrosion resistance of laser-cladded AlCoCrFeNi high-entropy alloy coatings with varying TiO_2_ content (0 wt.%, 0.5 wt.%, 1.0 wt.%, and 1.5 wt.%) in 3.5 wt.% NaCl solution. Optimal cladding parameters (1500 W, 40 mm/s, 11.9 g/min) were determined via orthogonal experiments. TiO_2_ promoted Ti-rich BCC2-phase precipitation, increased corrosion potential (from −1.4185 V to −0.6841 V), decreased corrosion current density (from 2.33 × 10^−4^ to 2.28 × 10^−6^ A/cm^2^), and enhanced charge-transfer resistance. XPS analysis demonstrated that TiO_2_ promoted the enrichment of FeO, Cr_2_O_3_, and TiO_2_ components in the passive film while reducing the Al_2_O_3_ fraction, leading to the formation of a dense and stable composite passive film that effectively inhibited chloride ion attack. In summary, an appropriate amount of TiO_2_ doping significantly enhances the corrosion resistance of laser-cladded AlCoCrFeNi HEA coatings, with the 1.5 wt.% addition being the best-performing among the investigated compositions.

## 1. Introduction

With the rapid development of critical industrial sectors such as marine engineering, petrochemicals, and nuclear power generation, various metal components often require long-term service under extreme conditions including high temperatures, high pressures, salt spray, or strong acidic/alkaline environments. Corrosive failure not only severely threatens equipment operational safety and stability but also poses significant economic losses and environmental risks. Although traditional protective alloys (e.g., stainless steel) exhibit certain corrosion resistance, they are prone to stress corrosion cracking in harsh environments with high salinity, failing to meet long-term protection requirements [1,2]. In recent years, high-entropy alloys (HEAs), leveraging unique effects such as “high-entropy mixing,” “lattice distortion,” “slow diffusion,” and “cocktail” mechanisms [3,4], have demonstrated superior performance compared to conventional single-element or minority-element alloys in mechanical properties [5], high-temperature stability [6,7], and corrosion resistance [8,9]. Particularly in complex corrosive environments, HEAs’ exceptional surface passivation capability has made them a research focus in corrosion protection [10]. Various techniques have been employed to fabricate HEA coatings, including magnetron sputtering and plasma spraying. While effective under specific conditions, these conventional methods face certain limitations in heavy-duty applications. For instance, magnetron sputtering is highly efficient for depositing thin films, but internal stresses can compromise coating adhesion as thickness increases [11]. Atmospheric plasma spraying typically forms a mechanical bond with the substrate and is prone to introducing higher porosity and in-flight oxidation depending on the specific deposition conditions [12]. In contrast, laser cladding utilizes a high-energy beam to rapidly melt the HEA precursors along with a thin layer of the substrate. Although it requires precise parameter optimization to control the dilution rate and thermal stress, this process inherently yields highly dense coatings with robust metallurgical bonding and minimal porosity, offering a highly suitable approach for thick, corrosion-resistant HEA applications [13]. In contrast, laser-cladding technology, utilizing a high-energy beam for rapid heating and solidification, effectively suppresses grain growth and produces high-performance coatings with metallurgical bonding to the substrate, high density, and uniform composition, offering a promising technical approach for enhancing the corrosion resistance of HEA coatings [14,15].

Among various high-entropy alloy systems, the AlCoCrFeNi family has garnered significant attention as a highly promising protective material. The inherent synergistic effect of its constituent elements, particularly the strong passivation capabilities of Cr and Al, facilitates the formation of robust multielement oxide films, providing exceptional thermal stability, mechanical strength, and corrosion resistance [16]. Recent studies have extensively investigated the electrochemical performance of AlCoCrFeNi alloys in aggressive chloride environments, demonstrating that their passivation behavior and pitting resistance are highly sensitive to their phase constitution and microstructural evolution [17]. For instance, researchers have highlighted the critical role of phase distribution and element segregation in determining the localized corrosion resistance of AlCoCrFeNi coatings [18]. However, in highly complex and aggressive corrosive environments, the intrinsic microgalvanic effects between multiple phases in single AlCoCrFeNi HEA coatings can still lead to localized passivation film breakdown, posing performance limitations. Introducing a well-designed second phase can further regulate its microstructure and enhance the overall corrosion performance. For example, Zhou et al. [19] found that HEA coatings reinforced with submicron-sized TiC particles achieved an optimal balance between wear resistance and corrosion resistance. Li et al. [20] incorporated small amounts of TiB_2_ ceramic particles into Al-Mg alloys supplemented with trace amounts of Sc and Zr elements, achieving not only improved mechanical strength but also significantly enhanced resistance to sensitized corrosion through multiphase synergistic effects. TiO_2_, with its excellent chemical stability, good mechanical properties, and unique passivation-promoting effects, is a highly promising ceramic-reinforced phase material [21]. Podelinska et al. [22] provides new insights into the process–structure relationships of nanocrystalline TiO_2_-based powders synthesized by extraction pyrolysis, hydrothermal, and sol methods. Perumal et al. [23] prepared composite materials (Al–5SiC, Al–5SiC–4Al_2_O_3_, Al–5SiC–4TiO_2_) by introducing Al_2_O_3_ and TiO_2_ ceramic particles into an Al6061 matrix, finding that TiO_2_ effectively reduces the electrochemical active surface area of the aluminum matrix and enhances the corrosion resistance of the matrix material. Consequently, TiO_2_ content not only significantly lowers the surface activity of the matrix and promotes the formation of a dense passivation film but also improves coating corrosion resistance by refining grain size and filling micropores [24,25]. However, TiO_2_ possesses excellent chemical stability and unique passivation-promoting characteristics. Although TiO_2_ has been widely utilized in organic primers and Al-based composites to reduce surface electrochemical activity, its behavior in high-temperature multicomponent HEA melts remains largely unexplored. Unlike inert physical additions, the high-temperature metallurgical interaction between TiO_2_ and the AlCoCrFeNi matrix during laser cladding introduces a complex synergistic mechanism—potentially altering local elemental segregation, oxygen distribution, and the selective growth of Ti-rich phases. Currently, there is a critical scarcity of systematic research comparing how such TiO_2_-driven microstructural evolution translates to the macroscopic corrosion resistance of laser-cladded HEA coatings. A concise comparison of representative Ti-bearing and ceramic/oxide-modified HEA coatings reported in previous studies is provided in Table 1.

Therefore, this study employed laser-cladding technology to prepare AlCoCrFeNi high-performance alloy (HEA) composite coatings doped with TiO_2_ at various mass fractions (0 wt.%; 0.5 wt.%; 1.0 wt.%; 1.5 wt.%). The research first systematically optimized laser-cladding process parameters through orthogonal experimental design, then focused on investigating the effects of different TiO_2_ content on the coating’s corrosion behavior in a 3.5 wt.% NaCl solution at room temperature. To elucidate the microscopic mechanisms, comprehensive analyses were conducted using X-ray diffraction (XRD), electron probe microanalysis (EPMA), and energy-dispersive spectroscopy (EDS) to examine TiO_2_’s regulatory effects on the coating’s phase composition, grain structure, and defects. Corrosion kinetics were evaluated via polarization curves and electrochemical impedance spectroscopy (EIS), while X-ray photoelectron spectroscopy (XPS) provided detailed insights into the chemical characteristics and formation mechanisms of the surface passivation film. This study aimed to clarify the relationship between TiO_2_ content and the evolution of the coating’s microstructure as well as its enhanced corrosion resistance, offering a solid theoretical foundation and technical guidance for the composition design and engineering applications of high-performance, corrosion-resistant, high-entropy alloy composite coatings.

## 2. Materials and Experiments

### 2.1. Preparation of Coating Materials

This experiment utilized AlCoCrFeNi HEA powder (particle size: 50–100 μm; purity: 99%) and nano-TiO_2_ powder (particle size: 25 nm; purity: 99.8%) as raw materials. The TiO_2_ powder was added to the HEA alloy powder at mass fractions of 0 wt.%, 0.5 wt.%, 1.0 wt.%, and 1.5 wt.% to prepare the following samples: pure AlCoCrFeNi HEA (Sample A); AlCoCrFeNi HEA doped with 0.5 wt.% TiO_2_ (Sample B); AlCoCrFeNi HEA doped with 1.0 wt.% TiO_2_ (Sample C); and AlCoCrFeNi HEA doped with 1.5 wt.% TiO_2_ (Sample D).

The mixture was transferred via a funnel into a clean container, and steel bearing balls were added as the grinding medium in a 1:3 ratio. To ensure uniform mixing, the gap between the container and the powder mixer was filled with medical-grade absorbent cotton to 95% capacity and sealed. The sealed container was then placed in a mixer and mechanically blended at 300 rpm for 48 h to achieve thorough homogenization of the composite powder. After mixing, the three powders were dried in a 100 °C oven for 4 h. Following natural cooling to room temperature, a high-entropy alloy composite coating was fabricated on the stainless steel substrate using laser-cladding technology.

### 2.2. Laser-Cladding Experiment

**Figure 1:**
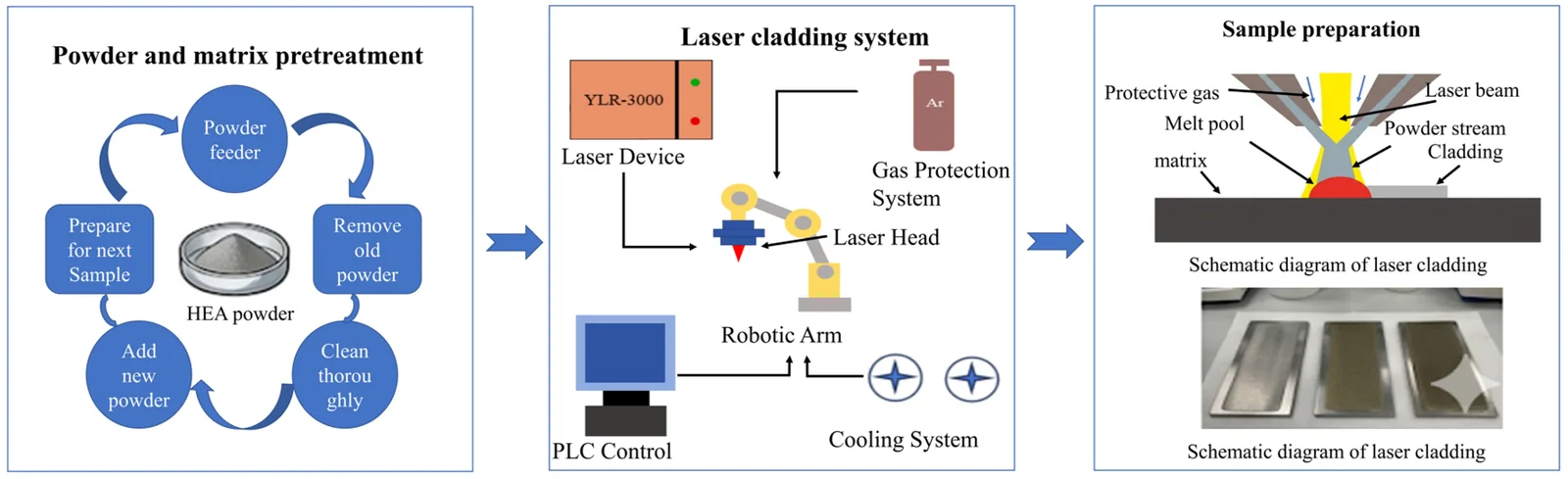
Process flow diagram of laser-cladding system.

A composite coating was prepared on the surface of a commercial 304L stainless steel substrate using a laser-cladding system (DMG MORI, Bielefeld, Germany, as shown in Figure 1). This system mainly consists of a high-power laser, a cooling system (DL20-900, China), a powder feeding and gas protection system, and a robotic arm. Before cladding, the substrate surface was successively ground with SiC sandpaper to remove rust and then ultrasonically cleaned with anhydrous ethanol. The laser-cladding procedure was as follows. Firstly, the initial powder was taken out from the container and placed in a drying oven for thorough drying (the drying process needs to be carried out at 100 °C for two hours). Then, the surface of the stainless steel substrate was polished with fine sandpaper and wiped with alcohol to remove impurities. When preparing coatings for samples A, B, C and D, the powder must be replaced after each cladding process (the original powder needs to be removed and the powder supply container cleaned before replacing with new powder to reduce experimental errors).

To determine optimal forming quality, this study employed a three-factor, four-level orthogonal experiment (see Table 2) to systematically evaluate the effects of laser power, scanning speed, and powder feed rate on the coating dilution rate and shape factor. After preparing single-layer coatings using 16 different parameter combinations, the dilution rates and shape factor values of these coatings were measured as output variables. The optimal process parameters were determined through range analysis, and subsequent test specimens were fabricated accordingly. High-purity argon gas (99.99%) at a flow rate of 10 L/min was continuously supplied throughout the cladding process as a protective gas to prevent oxidation of the high-temperature molten pool. The laser-cladding process utilized a coaxial powder-feeding geometry with a laser spot diameter of 3.0 mm and a focal position of +2 mm. To fabricate the macroscopic surface coatings, a continuous zig-zag scanning strategy was adopted with an adjacent track overlap ratio of 50%. The final fabricated specimens consisted of a single layer with an average total coating thickness of approximately 2 mm. Furthermore, following the cladding process, the actual Ti and O elemental distribution and relative content in the solidified coatings were verified via EDS area scanning to ensure consistency with the nominal TiO_2_ doping fractions.

### 2.3. Electrochemical Corrosion Behavior Testing

Using a wire-cutting machine (DK7740C, Suzhou, China), the AlCoCrFeNi high-entropy alloy (HEA) coating was cut into standard block specimens measuring 15 mm × 15 mm × 10 mm. To minimize surface scratches and obtain a flat observation surface, the working surfaces of the specimens were successively water-ground with silicon carbide (SiC) sandpaper of grit sizes 180, 500, 1000, and 2000, followed by mirror polishing using a 0.25 μm diamond polishing agent. The specimens were then ultrasonically cleaned with anhydrous ethanol and dried with cold air. Except for the designated working surface area, all other surfaces of the specimens were sealed with epoxy resin insulation.

The electrochemical tests were conducted at room temperature in a 3.5 wt.% NaCl solution using the multi-channel electrochemical workstation (CS310X, Wuhan Kesite Instrument Co, Ltd., Wuhan, China) and a standard three-electrode system: the coated sample served as the working electrode, the platinum mesh as the auxiliary electrode, and the saturated calomel electrode (SCE) as the reference electrode [26]. Prior to testing, the instrument was preheated at a constant temperature for 30 min to eliminate instrumental errors and environmental interference, followed by immersion of the reference electrode in the electrolyte for 15 min to achieve equilibrium. The sample was then left undisturbed in the solution while its open-circuit potential (OCP) was monitored for 1 h to establish a stable solid–liquid interface electrochemical equilibrium. Subsequently, electrochemical impedance spectroscopy (EIS) was performed with an AC sinusoidal perturbation amplitude of 10 mV and a frequency range of 10^−2^ Hz to 10^5^ Hz. Finally, a potentiodynamic polarization test was conducted with a scanning range of −0.5 V to 1.5 V (vs. OCP) at a rate of 0.5 mV/s to evaluate polarization potential, corrosion rate, and passivation region. To ensure the reliability and reproducibility of the experimental results, each sample was tested three times under identical experimental conditions. To further analyze the coating’s corrosion mechanism, a constant potential polarization test was performed by maintaining a specific voltage for a specified duration to obtain a stable passivation film. X-ray photoelectron spectroscopy (XPS) analysis of composition and oxidation states was carried out using the Axis-Ultra DLD-600W spectrometer (Kratos, Manchester, UK), equipped with a monochromatic Al Kα X-ray source (hv = 1486.6 eV) [27].

### 2.4. Microstructural Characterization

To systematically evaluate the phase composition, microstructural evolution, powder morphology and distribution, as well as surface failure characteristics after electrochemical corrosion of HEA coatings, this study employed a combination of various microscopic characterization techniques. X-ray diffraction (XRD, XRD-7000, Shimadzu, Kyoto, Japan) was used to analyze the crystal structure and phase composition of the high-entropy alloy powder and laser-cladding coatings with varying TiO2 content. Based on the diffraction results, the scanning range (2θ) was set between 10° and 90°. Field-emission scanning electron microscopy (FESEM, SIRION 200, FEI Company, Hillsboro, OR, USA) was utilized to observe the initial morphology and particle size distribution of the mixed powders, while electron probe microanalysis (EPMA, EPMA-8050G, Shimadzu Corporation, Kyoto, Japan) characterized the surface microstructure and cross-sectional metallurgical bonding interfaces of the coatings. Quantitative assessments were conducted on elemental diffusion behavior at the coating-substrate interface and the local segregation and distribution patterns of elements (e.g., Al, Co, Cr, Fe, Ni, Ti) within the coatings.

After completing the potentiodynamic polarization test, three-dimensional microscopic images of the corrosion regions on the coating surface were obtained using a super-depth-of-field 3D microscope (SuperView WT3000, Shenzhen, China). By extracting the two-dimensional cross-sectional contour curves of the corrosion pits, precise quantitative measurements of their geometric dimensions (maximum depth and opening width) were performed to visually assess the inhibitory effects of different TiO_2_ content levels on pitting corrosion lateral expansion and longitudinal penetration. To elucidate the passivation mechanism on the coating surface following the electrochemical reaction, constant potential polarization experiments were conducted to form a passivation film on the sample surface. Subsequently, X-ray photoelectron spectroscopy (XPS) analyses were carried out using an Axis-Ultra DLD-600W photoelectron spectrometer (Kratos, Manchester, UK), equipped with a monochromatic Al Kα X-ray source (hv = 1486.6 eV). Through the acquisition and fitting of core-level spectra for Al^2^p, Co^2^p, Cr^2^p, Fe^2^p, Ni^2^p, and Ti^2^p, the oxide composition and evolution of element valence states in the passivation film were thoroughly investigated.

## 3. Experimental Results and Analysis

### 3.1. Optimization of Laser-Cladding Process Parameters

**Figure 2:**
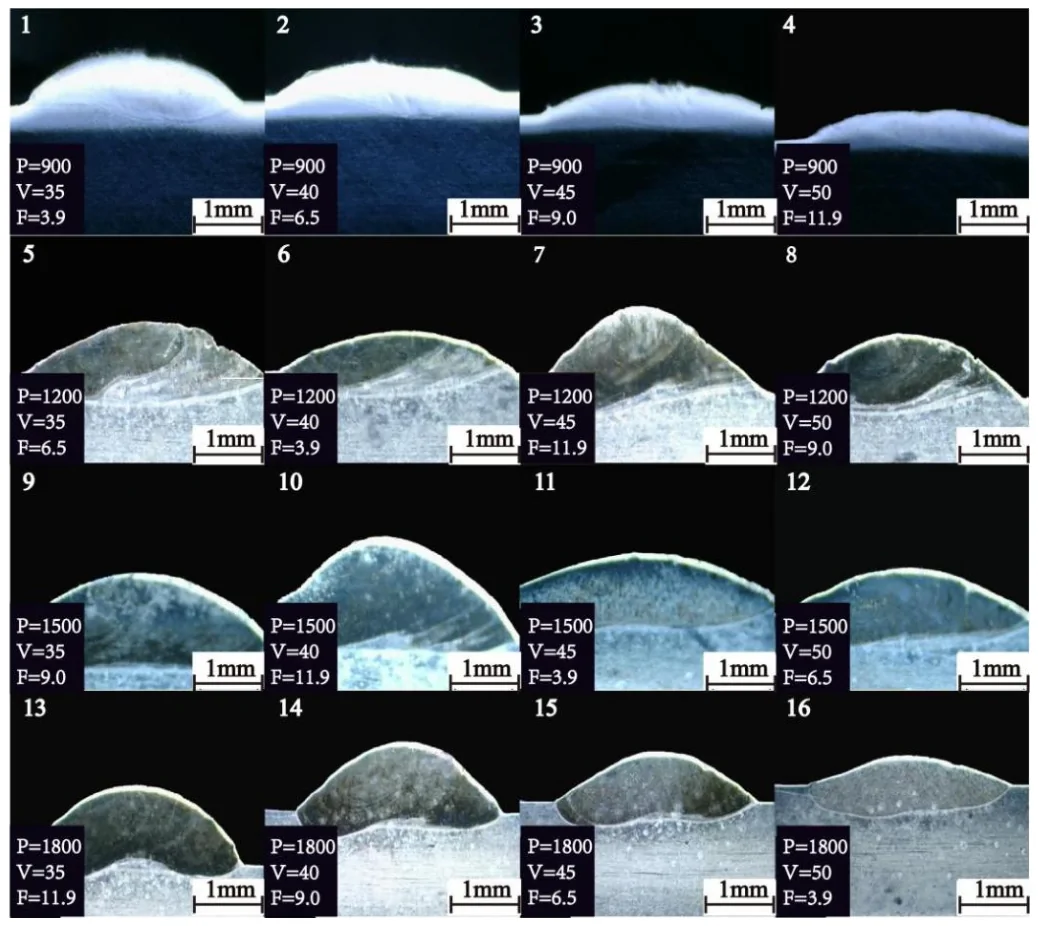
Cross-sectional morphology of a single-pass coating of AlCoCrFeNi HEA.

Strict control of scanning speed, powder feed rate, and laser power is crucial for obtaining high-quality laser-cladding coatings [28,29,30]. To identify the optimal process combination for preparing subsequent high-quality multilayer AlCoCrFeNi high-performance alloy (HEA) composite coatings, this study first prepared 16 sets of single-layer coating samples using a three-factor, four-level orthogonal experiment. As shown in the cross-sectional microstructure in Figure 2, the study systematically evaluated the metallurgical bonding status at the coating–matrix interface of all single-layer samples and examined the distribution of internal defects such as pores and cracks. Additionally, quantitative calculations were performed for key formation parameters (dilution rate and shape factor) for each coating group, with test data summarized in Table 3. Among these parameters, the dilution rate is a critical indicator of the metallurgical bonding strength between the coating and substrate as well as the retention rate of coating components. Excessively high dilution rates lead to excessive element migration from the substrate into the coating, compromising the initial design composition and inherent properties of the high-entropy alloy. Conversely, insufficient dilution rates fail to achieve adequate fusion depth, resulting in inadequate interfacial bonding strength [31]. Therefore, the core optimization principle of this study was to minimize the coating dilution rate while ensuring robust metallurgical bonding between the coating and substrate. All experimental data from the orthogonal experiment are presented in Table 3.

The microstructural characteristics of the cross sections of all samples were characterized and analyzed, with detailed observation of the metallurgical bonding interface between the coating and the substrate, as well as the distribution of internal defects such as pores and cracks. The cross-sectional morphology is shown in Figure 2. Additionally, quantitative measurements were performed for each coating group regarding key evaluation parameters (dilution ratio and shape factor).

To determine the importance of each process parameter on coating quality, identify key factors, and find the optimal combination of process parameters, this study employed range analysis to conduct an in-depth examination of the coating experimental data. Using range analysis, this paper evaluates the experimental data related to the coatings. Ki represents the sum of the experimental data from any column in the orthogonal table, while ki denotes the average value of *K_i_.*

(1) $$k_{i}=\frac{K_{i}}{s}$$

Here, s represents the number of occurrences at any given level in this column.

(2) $$\mathrm{Subsequently},\;\mathrm{the}\;\mathrm{range}\;R\;\mathrm{is}:\;R=\max\left\{k_{1},k_{2},k_{3},k_{4}\right\}-\min\left\{k_{1},k_{2},k_{3},k_{4}\right\}$$

The range data table for the orthogonal experiment was calculated using the aforementioned formula, as shown in Table 4.

To determine the primary and secondary influences of process parameters on coating quality, range analysis was employed to statistically process the orthogonal experimental data. The results indicated that the optimal ranges for laser power, scanning speed, and powder feed rate were 0.095, 0.075, and 0.0775, respectively. According to range analysis principles, larger range values signify more significant impacts on experimental outcomes. Consequently, the importance ranking of factors affecting coating dilution rate was: laser power (R = 0.095) > powder feed rate (R = 0.0775) > scanning speed (R = 0.075). As a critical indicator of metallurgical bonding between the coating and substrate and component retention rate, the dilution rate directly influences coating performance. Excessively high dilution rates lead to excessive diffusion of substrate elements into the coating, compromising its designed composition and inherent properties. Conversely, insufficient dilution rates may weaken the metallurgical bond strength and affect adhesion stability [32]. Therefore, the core objective of the experimental optimization was to minimize the dilution rate while ensuring optimal adhesion between the coating and the substrate. The dilution rate of sample group 10 was 0.16, the lowest among all samples. Based on the range analysis results and the evaluation of cross-sectional defects and formation quality across all 16 sample groups, the optimal process parameters for preparing this HEA coating were determined: laser power of 1500 W, scanning speed of 40 mm/s, and powder feed rate of 11.9 g/min. This optimized parameter set was subsequently applied directly to the fabrication of all multilayer modified composite coatings.

### 3.2. Microstructural Analysis of the Coating

**Figure 3:**
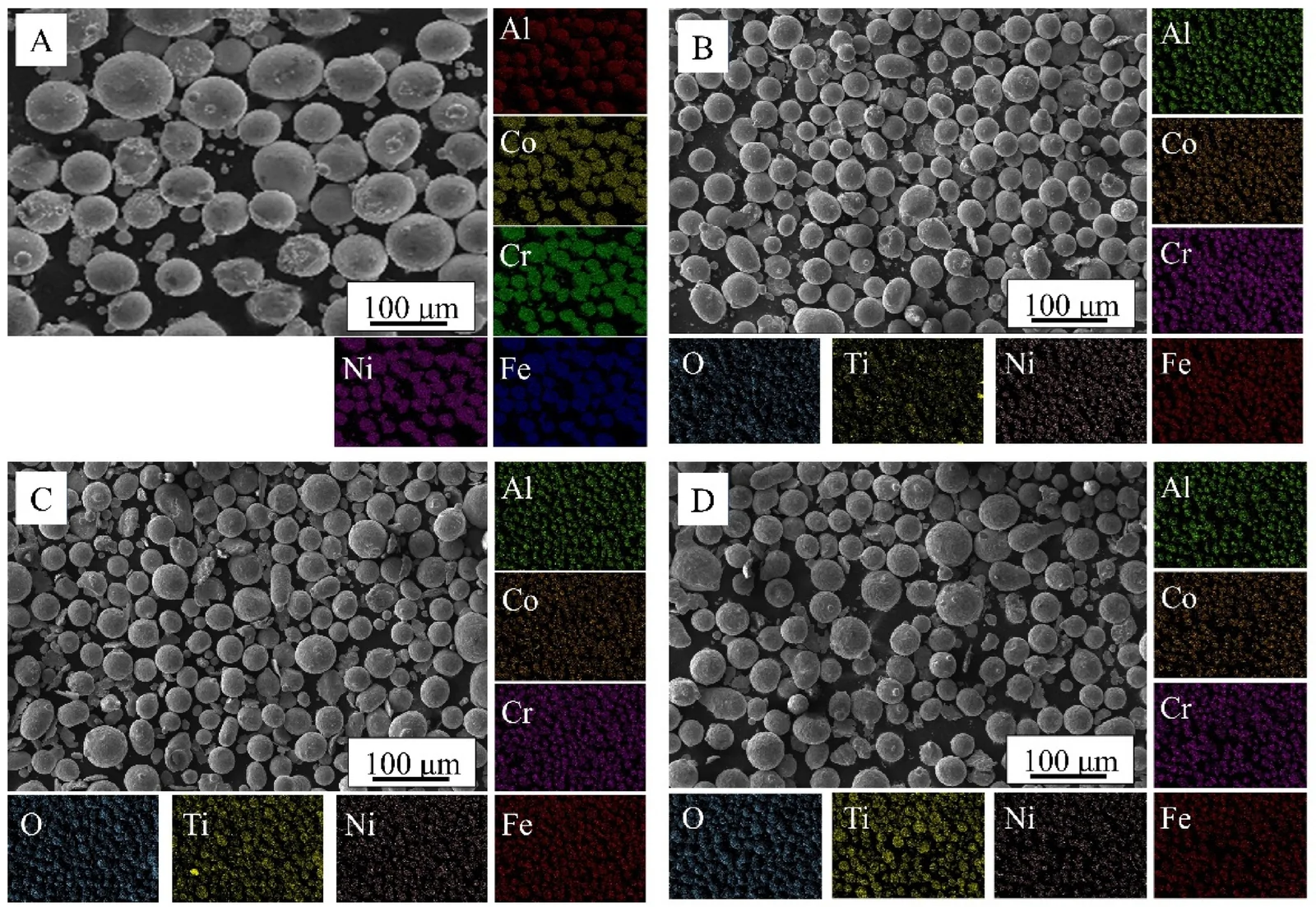
Scanning electron microscope images of AlCoCrFeNi HEA powders doped with different TiO_2_ content: (**A**) 0 wt.%; (**B**) 0.5 wt.%; (**C**) 1 wt.%; (**D**) 1.5 wt.%.

Figure 3 presents the SEM morphologies and energy-dispersive spectroscopy (EDS) elemental maps of the original AlCoCrFeNi high-entropy alloy powder before mechanical mixing and the TiO_2_-containing powders after mechanical mixing. Compared with the original powder, the mechanically mixed powders exhibit slight particle deformation and a modest increase in surface irregularity. Nevertheless, most of the AlCoCrFeNi particles remain predominantly spherical, with no obvious large-scale flattening, severe fragmentation, or extensive cold welding observed under the present mixing conditions. The overall particle size range also appears comparable to that of the original powder based on the SEM observations. In addition, the EDS elemental maps show that the constituent elements are relatively homogeneously distributed, while Ti and O are detected throughout the TiO_2_-containing powder mixtures, indicating effective incorporation and dispersion of TiO_2_. The predominantly spherical morphology of the mixed powders is favorable for stable powder feeding and subsequent melting during the laser-cladding process.

According to the Hume-Rothery principle, the formation of phase structures in high-entropy alloy systems is determined by the relative strength of chemical affinities among the alloy elements [33]. However, differences in chemical affinities among multiple elements lead to distinct phase structures in different systems. The VEC values for AlCoCrFeNi HEA were calculated using Equation (3).

(3) $$\mathrm{VEC}=\sum_{i=1}^{n}c_{i}{\left(\mathrm{VEC}\right)}_{i\;}$$

In the formula, c_i_ denotes the atomic percentage of the i-th element, while (VEC)_i_ represents the valence electron concentration of the i-th element. All data required for this study are available in the table [34]. Substitution of the data into Equation (3) yields the following result: the VEC value for AlCoCrFeNi HEA is 7.2, indicating that this alloy system may exhibit a tendency to form FCC + BCC or BCC-related solid solution structures. However, upon addition of TiO_2_, the system ceases to be a single metal solid solution and instead constitutes a composite structure comprising a metal matrix phase, a Ti-enriched phase, and oxide/reaction products [35]. Therefore, the phase composition of TiO_2_-added samples cannot be predicted solely based on traditional VEC criteria, but should primarily be determined through XRD analysis.

**Figure 4:**
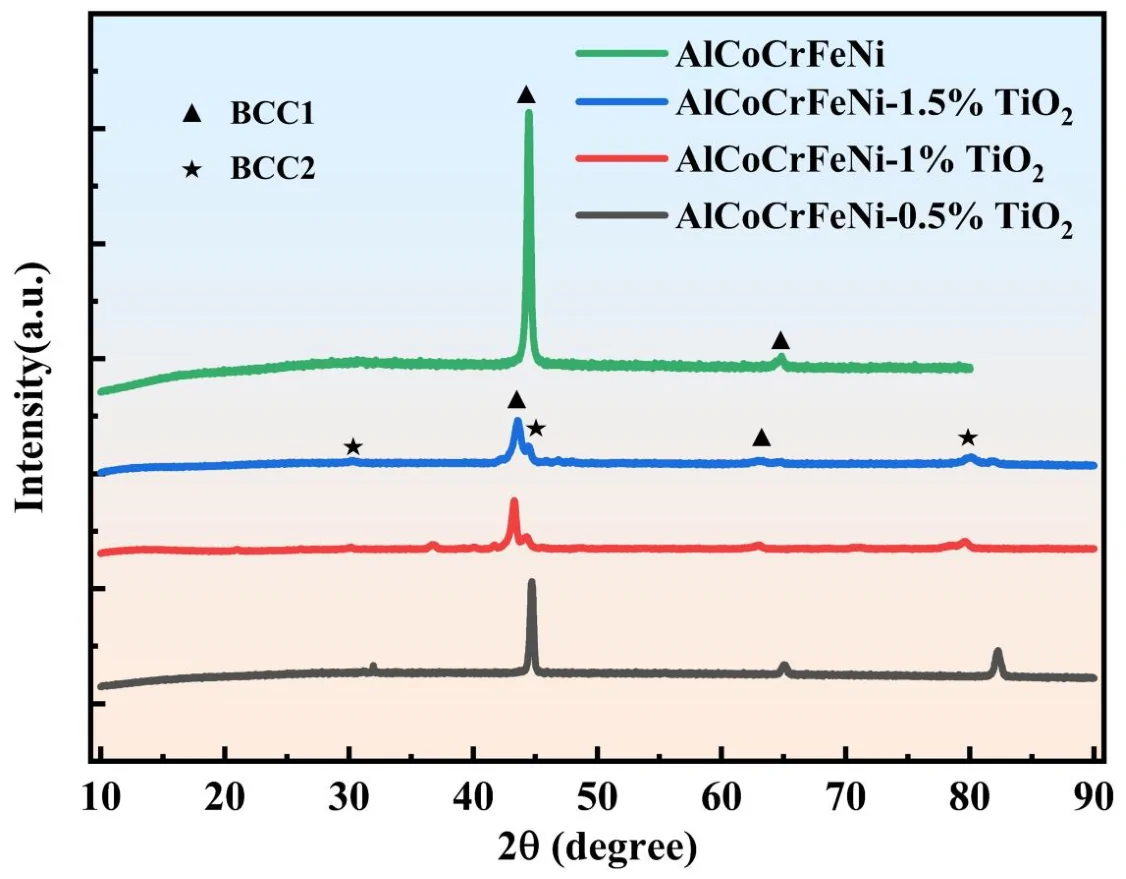
XRD patterns of AlCoCrFeNi HEA coatings with different TiO_2_ content (0 wt.%, 0.5 wt.%, 1.0 wt.% and 1.5 wt.%).

Figure 4 shows the XRD spectra of AlCoCrFeNi HEA coatings with different TiO_2_ content (0 wt.%, 0.5 wt.%, 1.0 wt.%, and 1.5 wt.%). Analysis indicates that all samples maintain a crystal structure dominated by the body-centered cubic (BCC) phase. Precise calibration of the diffraction peak positions reveals distinct peaks at approximately 44.6° and 65.0° for the TiO_2_-free substrate (AlCoCrFeNi), corresponding to the crystal planes of the BCC_1_ structure. This confirms that the high-entropy alloy matrix possesses a typical single-phase BCC crystal structure, typically attributed to lattice distortion effects caused by the large atomic radius of aluminum. With the addition of TiO_2_, the main peak of the BCC phase shifts significantly toward lower angles (left shift). According to Bragg’s law, this phenomenon indicates an increase in the lattice constant d due to the solid solution incorporation of Ti atoms into the BCC matrix lattice. Notably, no distinct independent TiO_2_ oxide diffraction peaks were observed in the XRD spectra, suggesting that TiO_2_ likely decomposed during the cladding process, facilitating Ti incorporation into the matrix lattice or participation in the formation of secondary phases.

While a semiquantitative estimation of the diffraction peak areas indicates a progressive increase in the intensity of the secondary peaks with higher TiO_2_ content (suggesting a TiO_2_-induced phase separation trend), the inherently broad and overlapping nature of these peaks restricts precise quantitative extraction of phase fractions and lattice parameters. Therefore, the identification of this secondary phase as a distinct “BCC2” structure is currently considered a tentative assignment rather than an established crystallographic fact. Definitive confirmation of its exact crystal structure and precise lattice parameters would necessitate future high-resolution characterizations, such as transmission electron microscopy (TEM) or electron backscatter diffraction (EBSD). This phase separation phenomenon not only modulates the stress distribution in the matrix but also provides a theoretical basis for enhancing coating performance at the microstructural level.

Analysis of XRD data indicates that with increasing TiO_2_ content, more secondary phases (i.e., BCC2) form on the surface of the AlCoCrFeNi HEA coating. This suggests that the addition of TiO_2_ not only induces significant phase separation within the alloy but also exacerbates lattice distortion in the matrix [36].

**Figure 5:**
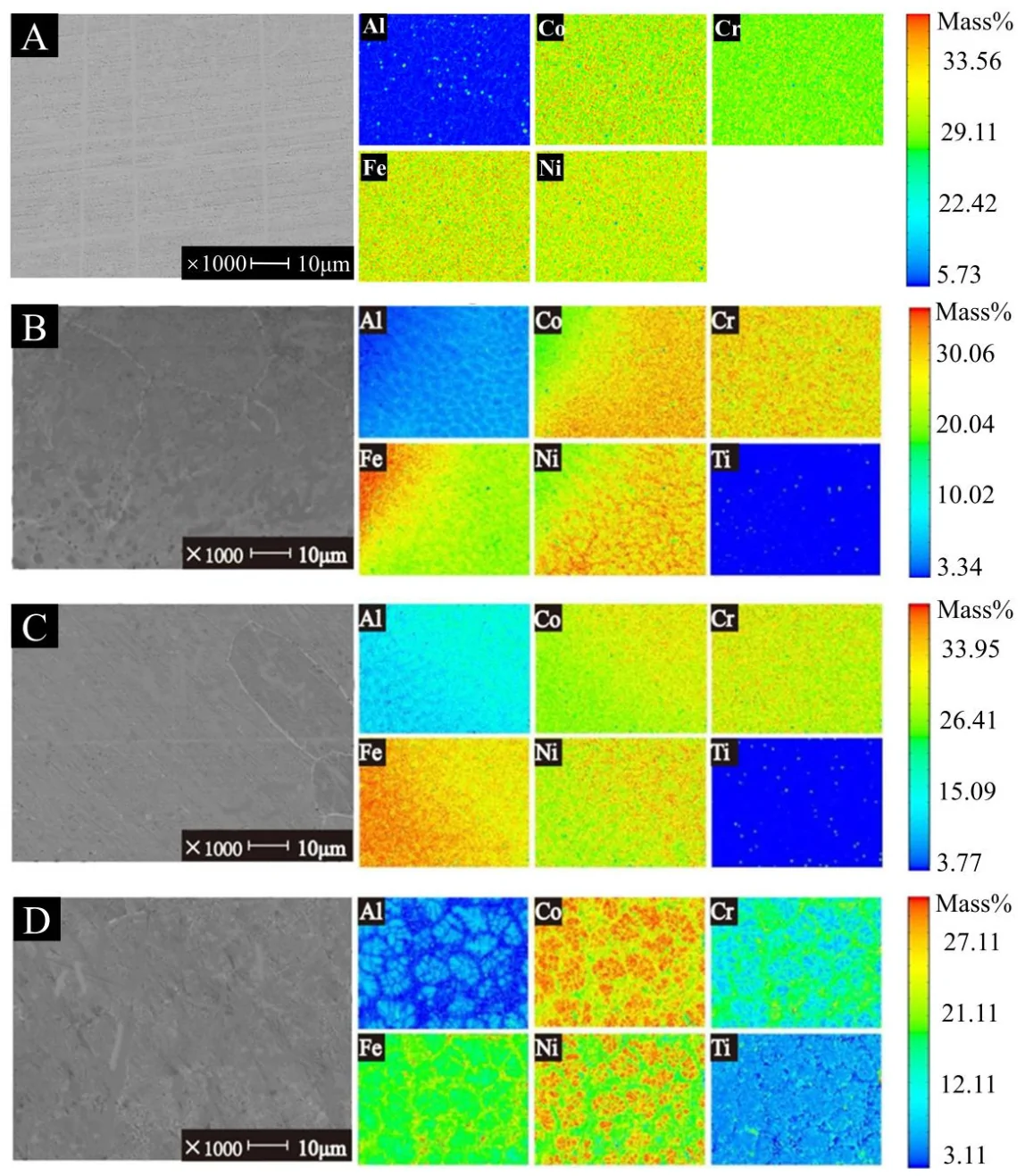
Surface scanning images of AlCoCrFeNi HEA coating samples with different TiO_2_ content: (**A**) 0 wt.%; (**B**) 0.5 wt.%; (**C**) 1 wt.%; (**D**) 1.5 wt.%.

Figure 5 illustrates the microstructural evolution of AlCoCrFeNi HEA coatings under varying TiO_2_ content levels. As the TiO_2_ content increases from 0 wt.% to 1.5 wt.%, the phase separation characteristics of the coating become more pronounced, with the microstructure primarily consisting of an Ni–Al-rich ordered phase, a Co–Cr–Fe-rich matrix phase, and localized Ti–Cr-rich regions. Notably, the formation of the Ni–Al-rich ordered phase is primarily attributed to the strong negative mixing enthalpy between Ni and Al and their tendency toward ordering. However, the Ti released or introduced by TiO_2_ content does not entirely enter the Ni–Al ordered phase. Instead, it participates in competitive element distribution within the multicomponent system during rapid solidification.

From a thermodynamic perspective, the large atomic radius of titanium (Ti) significantly increases the atomic size mismatch and lattice distortion energy in the system [37]. To reduce local elastic energy and chemical free energy, the alloy system tends to optimize energy through element segregation and phase separation. On one hand, the strong interaction between nickel (Ni) and aluminum (Al) promotes the formation of an ordered Ni–Al-rich phase. On the other hand, Ti, as a typical BCC-stabilizing element, tends to accumulate in BCC-structured regions when its local concentration exceeds the matrix’s solid solution capacity. Meanwhile, chromium (Cr), with its high solid solution stability in the BCC phase, readily co-accumulates with Ti, forming secondary Ti–Cr-rich BCC regions. Thus, the presence of Ti–Cr-rich phases does not indicate the strongest negative mixing enthalpy between Ti and Cr, but rather results from the combined effects of elemental interactions, crystal structure stability, atomic size mismatch, and solute redistribution under rapid-solidification conditions in multicomponent high-entropy alloys [38].

Regarding the Ti–Cr-rich regions observed in the microstructural maps, they are tentatively correlated with the emerging secondary diffraction peaks in the XRD patterns. It should be noted that without higher-resolution crystallographic evidence, assigning these specific element-segregated regions directly to the proposed BCC2 phase remains a suggested microstructural evolution rather than a definitive proof. These findings are consistent with the thermodynamic principles governing how Ti stabilizes the BCC/B2 phases and promotes compositional segregation in AlCoCrFeNiTi high-entropy alloys. From a solidification kinetics perspective, fine TiO_2_ particles serve as heterogeneous nucleation centers during melt pool solidification, reducing the critical nucleation energy for local phase precipitation. Additionally, with its large solute atoms, Ti exhibits low diffusion rates in the high-entropy matrix. Under rapid-solidification conditions, its long-range diffusion is restricted, leading to solute accumulation near dendritic interstices or phase boundaries. This ultimately facilitates the localized expansion of Ti-rich regions and enhances the definition of phase boundary contours [39].

In summary, the regulation of the microstructure in AlCoCrFeNi HEA coatings by TiO_2_ content does not stem solely from the mixing enthalpy effects between individual elements, but rather results from a combination of factors: Ni–Al ordering, Ti’s BCC stabilizing effect, Cr’s tendency to dissolve into the BCC phase, lattice distortion, and solute redistribution during rapid solidification. This mechanism provides a more coherent explanation for the synergistic evolution observed: namely the enhanced Ni–Al ordered phase and the formation of Ti–Cr BCC_2_ regions in TiO_2_-doped coatings.

**Figure 6:**
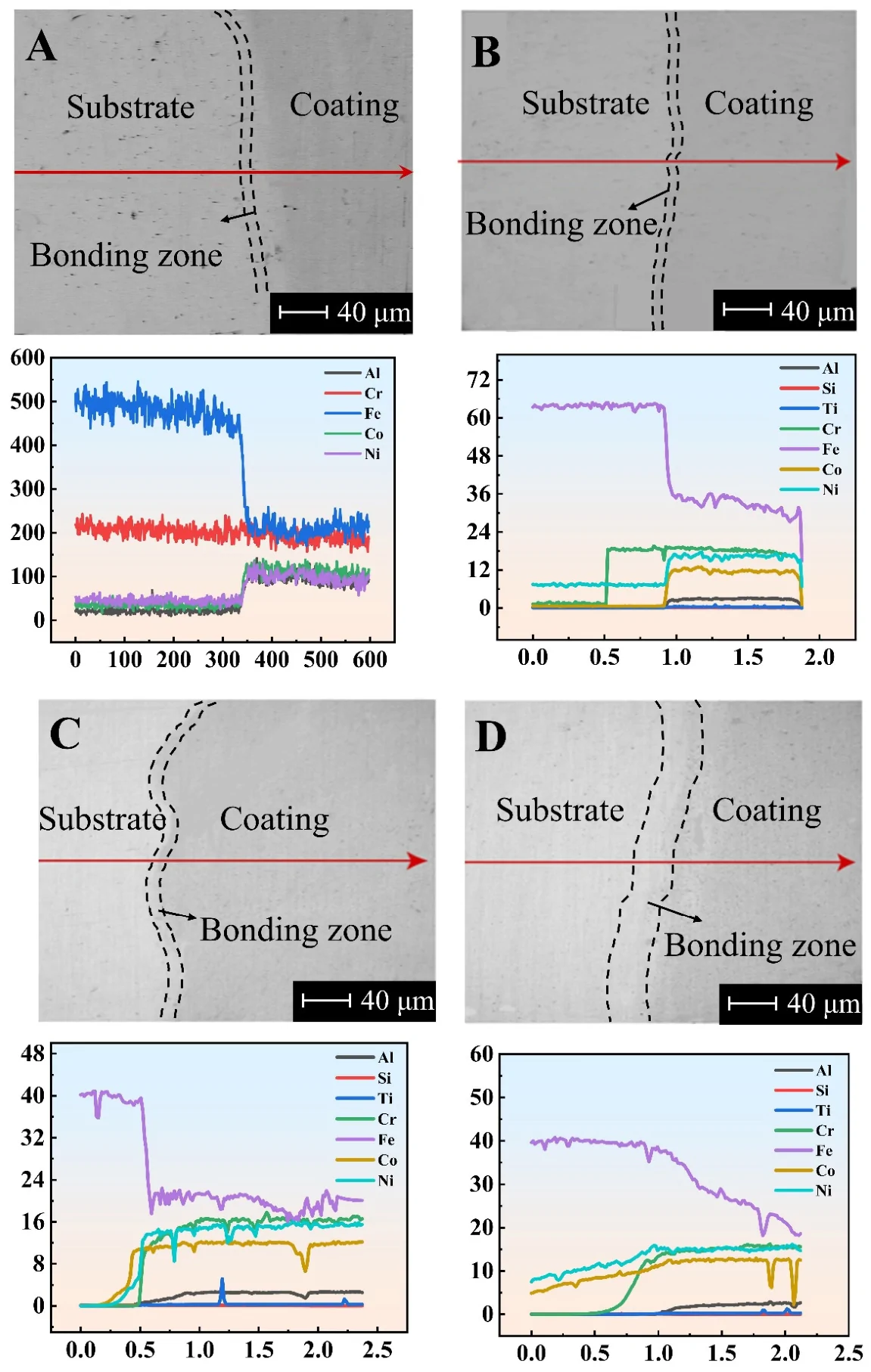
Cross-sectional line scan images of AlCoCrFeNi HEA coatings on stainless steel substrates with different TiO_2_ content: (**A**) 0 wt.%; (**B**) 0.5 wt.%; (**C**) 1 wt.%; (**D**) 1.5 wt.%.

The line scan results in Figure 6 reveal the microstructural evolution between the substrate and the AlCoCrFeNi HEA coating, as well as between the substrate and the TiO_2_-doped AlCoCrFeNi HEA coating, within the sample cross section. As shown, a distinct transition zone exists between the substrate (light-colored area) and the coating (dark-colored area). Line scan analysis confirms significant element mutual diffusion: silicon (Si) from the substrate migrates toward the deposit layer, while chromium (Cr), nickel (Ni), and titanium (Ti) from the coating diffuse into the substrate. SEM images demonstrate a continuous grain structure at the interface of the TiO_2_-doped AlCoCrFeNi HEA coating, collectively demonstrating the formation of a metallurgical bond between the two layers.

Furthermore, as the TiO_2_ content increases, the width of the element diffusion zone increases significantly (most notably in Figure 6D), indicating further enhancement of interfacial bonding. This mechanism can be attributed to three factors: first, TiO_2_ content as a ceramic phase improves the melt pool’s absorption rate of laser energy, thereby raising its temperature and prolonging its duration, which facilitates thermal diffusion of solute atoms; second, titanium exhibits high chemical reactivity that effectively reduces the interfacial tension between the slag and the matrix metal, enhancing wettability; and finally, TiO_2_ particles refine grains near the interface within the melt pool, consolidating metallurgical bonding by increasing the effective contact area between interfaces.

### 3.3. Analysis of the Corrosion Behavior of the Coating

**Figure 7:**
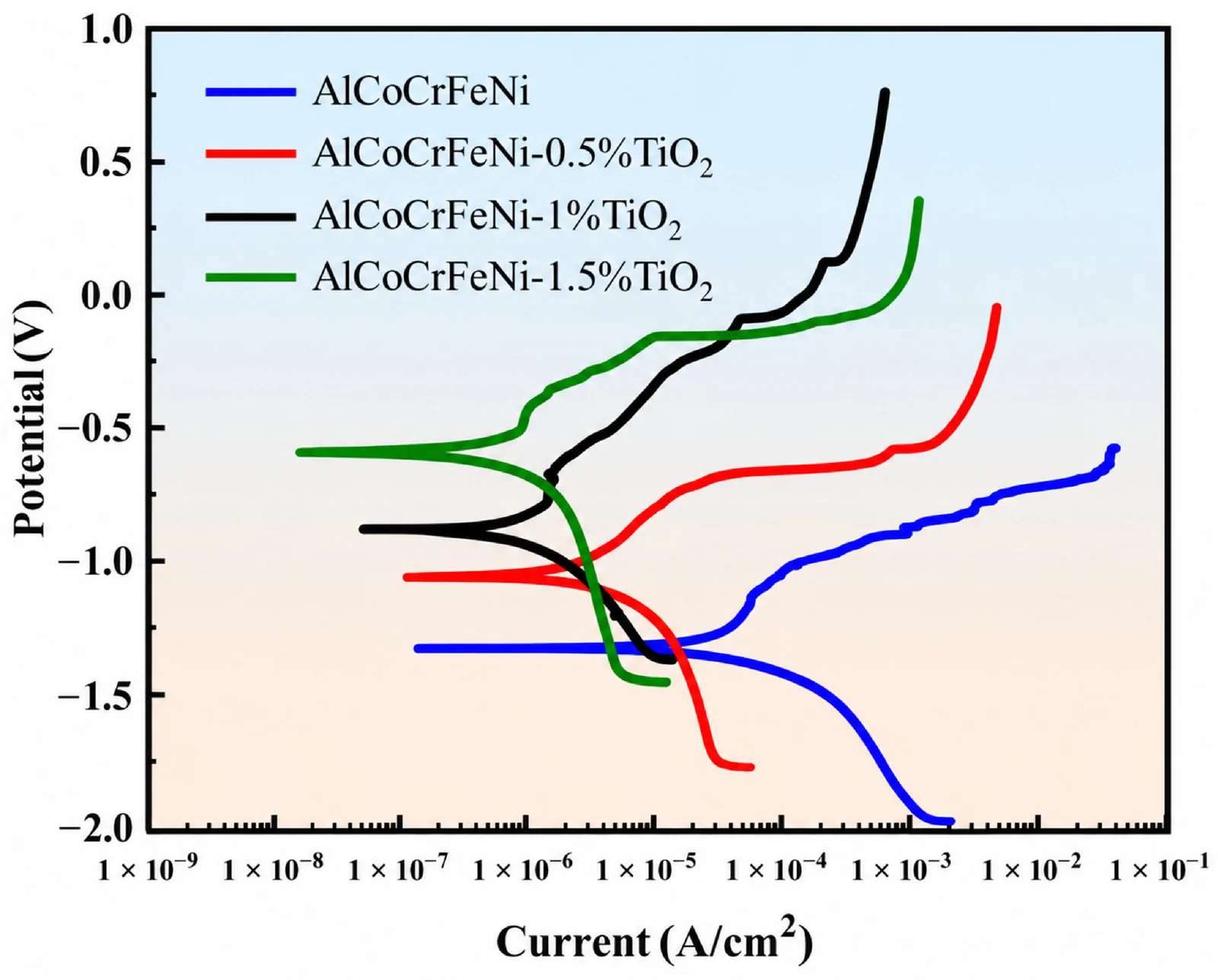
Dynamic potential polarization curves of AlCoCrFeNi HEA coatings with varying TiO_2_ content.

To evaluate the effect of different TiO_2_ content levels on the corrosion resistance of the AlCoCrFeNi coating, a potentiodynamic polarization test was conducted in a 3.5 wt.% NaCl solution (Figure 7). Tafel fitting results (Table 5) showed that the Ecorr value of the AlCoCrFeNi HEA coating was the lowest; however, the addition of TiO_2_ significantly increased the Ecorr value, with further elevation as TiO_2_ content increased. Notably, I_corr_ decreased with increasing TiO_2_ content, indicating that the positive shift in E_corr_ and the reduction in I_corr_ were primarily due to strong inhibition of the anodic reaction (metal dissolution), resulting in a denser passivation film. Conversely, the AlCoCrFeNi HEA exhibited the highest current density, and the cathodic branch shifted toward lower current densities as TiO_2_ content increased, driving the corrosion potential toward the noble metal potential [40,41]. Despite enhanced cathodic activity, the coating maintained a low overall corrosion current density (I_corr_) owing to TiO_2_-induced microstructural optimization.

The mechanism behind the elevated E_corr_ is primarily attributed to the dual effects of TiO_2_. On one hand, its electrocatalytic effect inhibits the anodic reaction, thermodynamically driving a positive shift in the self-corrosion potential. On the other hand, TiO_2_-induced grain refinement facilitates rapid formation of a dense surface passivation film, which not only activates the cathodic reaction but also enhances the physical barrier function, ensuring the material’s long-term chemical stability at higher potentials.

**Figure 8:**
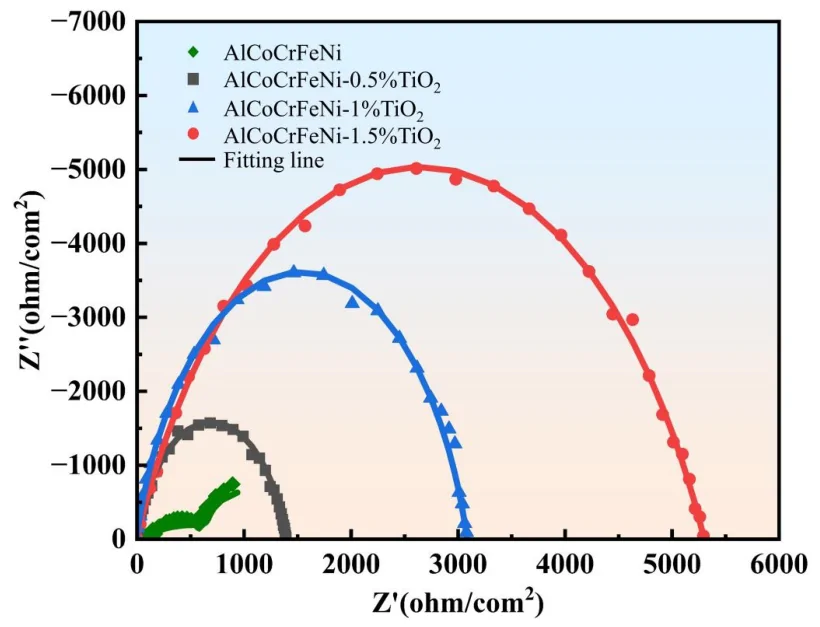
Electrochemical impedance spectroscopy and equivalent circuit of the coating.

To investigate the interface electrochemical behavior and protective mechanism of the passivation film on the coating surface in depth, the study employed electrochemical impedance spectroscopy (EIS) technology [42]. Figure 8 displays the Nyquist plots of the samples along with their corresponding equivalent circuit models. In the Nyquist plots, the capacitance arc radius directly reflects the protective efficacy of the passivation film: a larger capacitance arc radius indicates higher passivation film density, greater charge-transfer resistance, and more pronounced shielding protection against the substrate [43,44]. As shown in Figure 8, the capacitance arc radii for samples with different TiO_2_ concentrations were: 1.5 wt.% TiO_2_ > 1.0 wt.% TiO_2_ > 0.5 wt.% TiO_2_ > 0 wt.% TiO_2_. These results align with potentiodynamic polarization test data, further confirming that TiO_2_ enhances the corrosion resistance of the AlCoCrFeNi high-entropy alloy (HEA), with the 1.5 wt.% TiO_2_-doped coating exhibiting the best corrosion resistance among the tested groups. The EIS data were analyzed using ZView 3 software, employing the equivalent circuit model comprising “solution resistance (Rs)—passivation film/substrate charge transfer resistance (Rct)—constant phase element (CPE)” [45,46].

**Figure 9:**
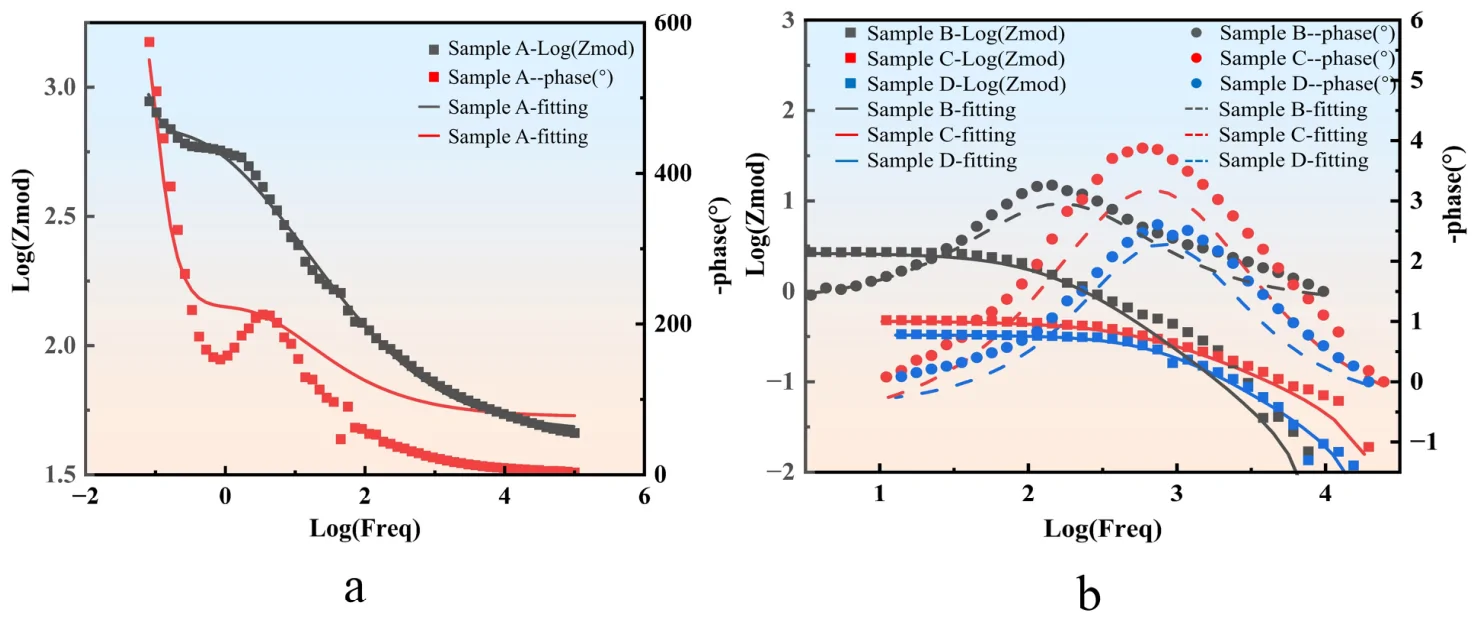
Bode plots and corresponding fitting curves of AlCoCrFeNi HEA coatings with different TiO_2_ content: (**a**) sample A (0 wt.% TiO_2_); (**b**) samples B–D, containing 0.5, 1.0, and 1.5 wt.% TiO_2_, respectively.

Figure 9 presents the Bode plots and corresponding fitting results of the AlCoCrFeNi HEA coatings with different TiO_2_ content. The impedance modulus generally decreases with increasing frequency, while the phase-angle curves exhibit distinct frequency-dependent responses, indicating the capacitive characteristics of the coating–electrolyte interface. Samples B–D show noticeable differences in both the impedance modulus and phase-angle response, suggesting that TiO_2_ content affects the interfacial electrochemical behavior of the coatings. The fitted curves generally agree with the experimental data, supporting the applicability of the selected equivalent-circuit model for describing the electrochemical response of the coatings.

Table 6 summarizes the EIS data-fitting results for AlCoCrFeNi HEA coatings with varying TiO_2_ content levels. The fitting parameters indicate that the Rct value of the 0.5 wt.% TiO_2_ sample is 1802.57 Ω·cm^2^, while that of the 1.0 wt.% TiO_2_ sample rises to 3347.01 Ω·cm^2^, and further increases to 4821.00 Ω·cm^2^ for the 1.5 wt.% TiO_2_ sample, demonstrating a clear upward trend in Rct values with increasing TiO_2_ content. According to EIS theory, the Rct value directly reflects the passivation film’s ability to hinder charge transfer: higher Rct values indicate greater resistance to electron penetration through the passivation film for redox reactions on the substrate, effectively suppressing charge exchange between the substrate and the corrosive medium and significantly enhancing the coating’s corrosion resistance [47]. Comprehensive analysis of EIS measurements and fitting results shows that in a 3.5 wt.% sodium chloride corrosive medium, the corrosion resistance of the AlCoCrFeNi HEA coating improves markedly with increasing TiO_2_ content levels.

**Figure 10:**
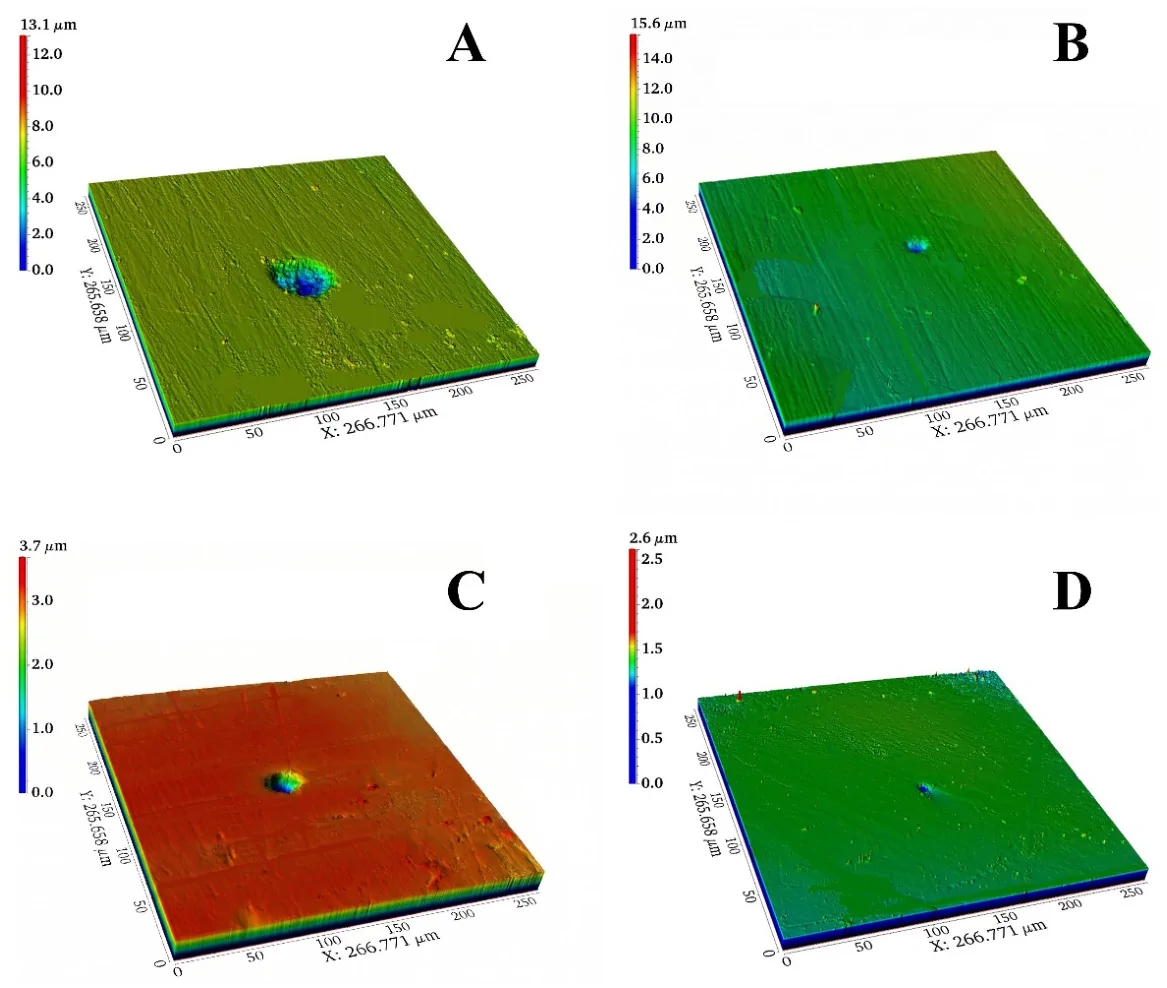
Three-dimensional morphological images of corrosion pits in AlCoCrFeNi HEA coatings with varying TiO_2_ content: (**A**) 0 wt.%; (**B**) 0.5 wt.%; (**C**) 1 wt.%; (**D**) 1.5 wt.%.

Figure 10 shows the surface morphology obtained via super-depth-of-field microscopy, providing an intuitive analysis of how different TiO2 content levels affect the pitting corrosion resistance of the AlCoCrFeNi HEA coating. The corrosion pits in the AlCoCrFeNi HEA coating exhibit a “wide and deep” characteristic, with large pit openings surrounded by distinct accumulation of corrosion products or secondary dissolution traces, indicating that the coating is prone to extensive localized pitting corrosion expansion in a 3.5% NaCl solution. In contrast, the AlCoCrFeNi HEA coating doped with 0.5% TiO_2_ demonstrates improved macroscopic flatness, with fewer large shallow pits, but the appearance of pronounced deep-pit defects. However, as the TiO_2_ content increases further, the volume of corrosion pits significantly decreases. Notably, in the 1.5% TiO_2_ coating, only minimal residual pitting corrosion is observed, resulting in the most uniform three-dimensional morphology (with a maximum height difference of merely 2.6 μm) and enhanced corrosion resistance.

To quantitatively evaluate the pitting resistance, geometric data were extracted from the 3D optical profilometry. The quantitative analysis indicated that as the TiO_2_ content increased, the localized corrosion damage was significantly mitigated. Specifically, the maximum pit depth drastically decreased from approximately 13.1 μm in the undoped AlCoCrFeNi coating to merely 2.6 μm in the 1.5 wt.% TiO_2_ sample. Concurrently, the average opening diameter of the corrosion pits correspondingly shrank. The group doped with 1.5 wt.% TiO_2_ demonstrated the smallest corrosion pit depths and minimum volumetric material loss, exhibiting the highest pitting resistance among the investigated compositions.

**Figure 11:**
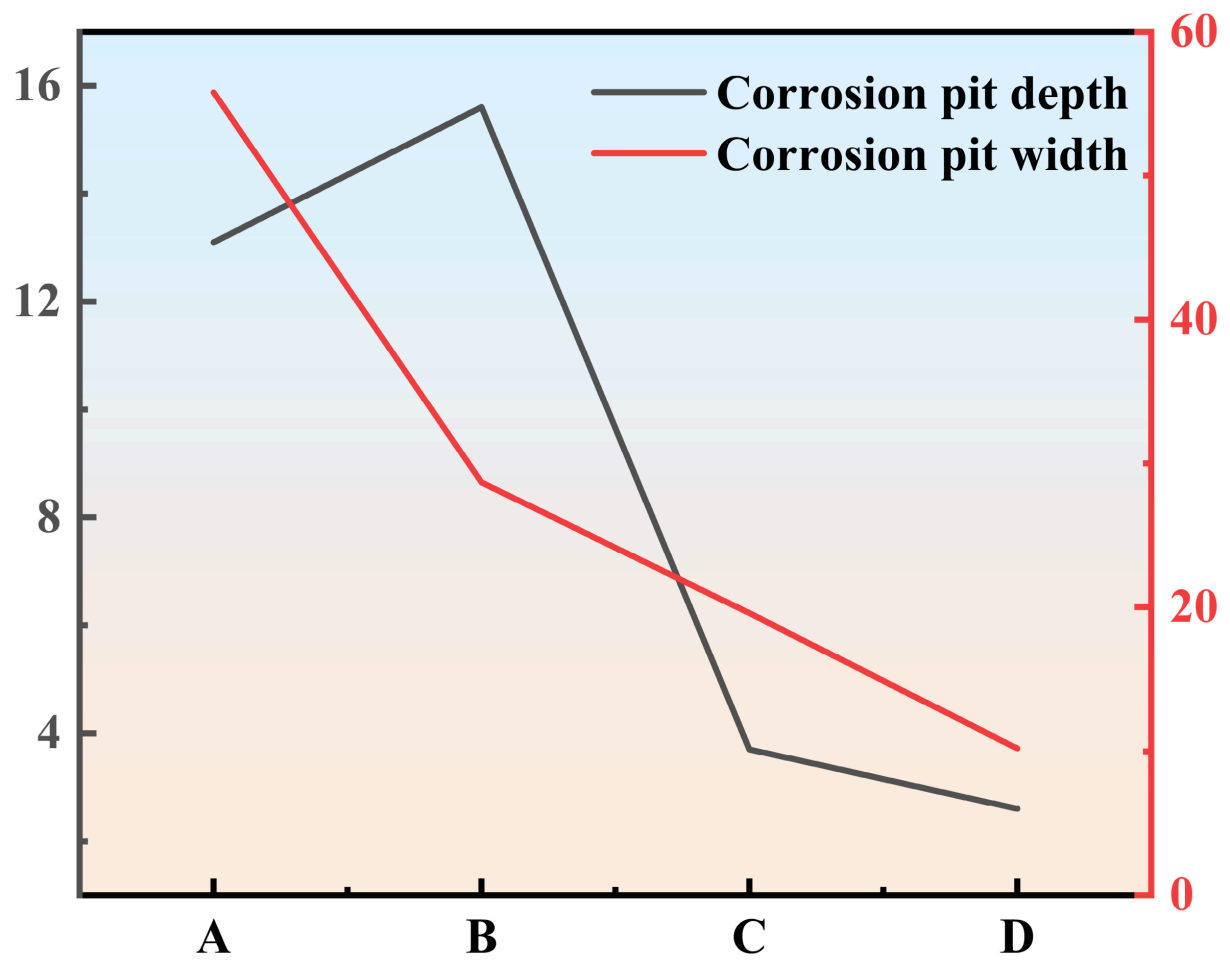
Comparison of corrosion pit depth and width of AlCoCrFeNi HEA coatings with different TiO_2_ content: A. 0 wt.%; B. 0.5 wt.%; C. 1.0 wt.%; D. 1.5 wt.%.

As shown in Figure 11, the corrosion pit dimensions vary significantly with increasing TiO_2_ content. The pit width decreases continuously from Sample A to Sample D, while the pit depth shows a slight increase from Sample A to Sample B, followed by a pronounced decrease for Samples C and D. In particular, Sample D exhibits the smallest pit depth and width, indicating that 1.5 wt.% TiO_2_ most effectively suppresses both the lateral propagation and vertical penetration of pitting corrosion. These results are consistent with the electrochemical measurements, further confirming the improved corrosion resistance of the TiO_2_-modified AlCoCrFeNi HEA coatings.

### 3.4. Passivation Film Composition Analysis

**Figure 12:**
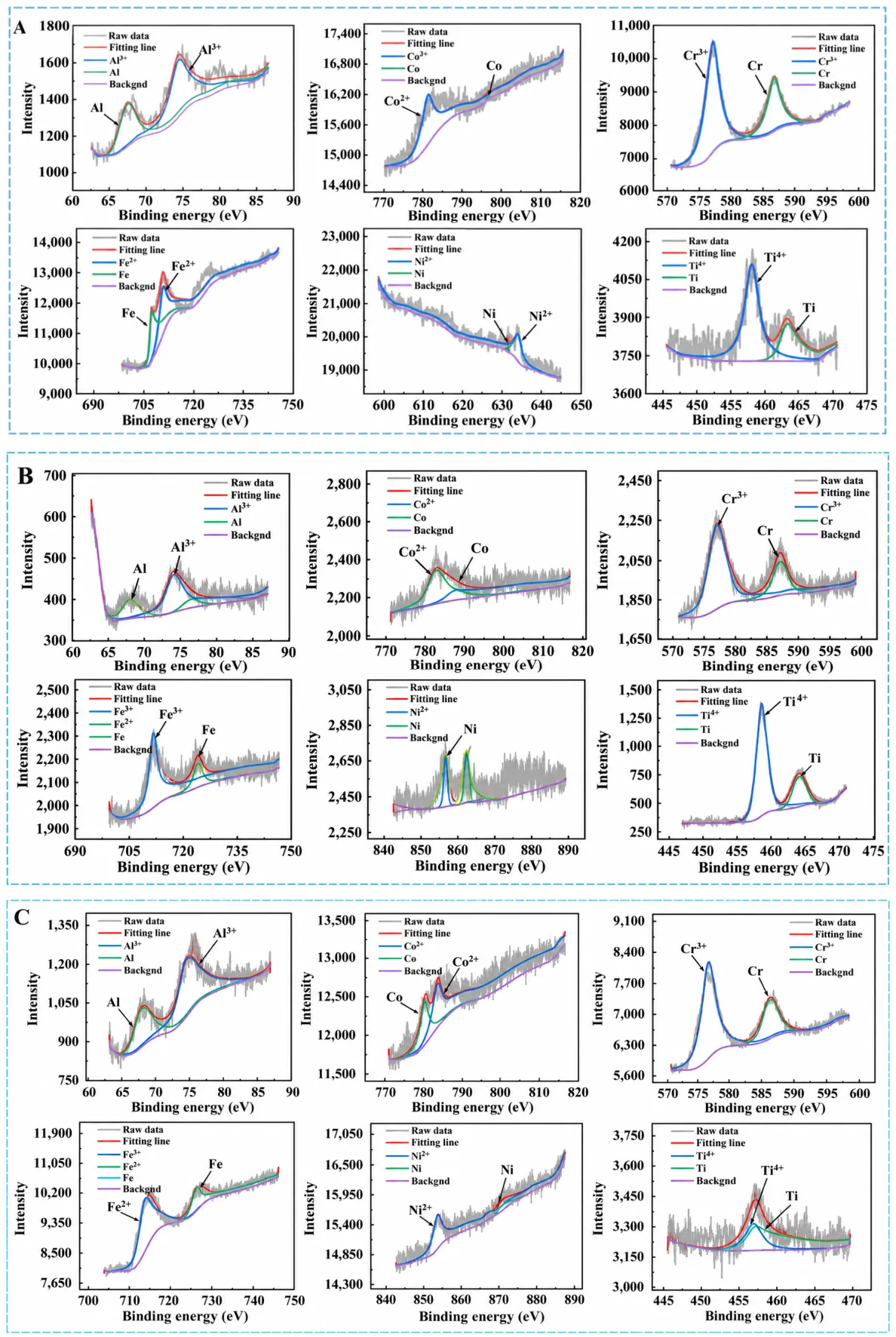
XPS spectra of passivation films on AlCoCrFeNi HEA coatings with different TiO_2_ content: (**A**) 0.5 wt.%; (**B**) 1 wt.%; (**C**) 1.5 wt.%.

In the electrochemical corrosion experiments of AlCoCrFeNi HEA coatings, the formed passivation film on the surface serves as the critical carrier for analyzing corrosion resistance mechanisms and elucidating protective mechanisms. Its chemical composition and microstructural characteristics directly determine the coating’s corrosion resistance performance [48]. To systematically investigate the regulatory effects of different TiO_2_ content levels on the composition of the HEA coating passivation film and further elucidate the intrinsic mechanism of TiO_2_-enhanced corrosion resistance, this study conducted XPS analyses on three groups of HEA coatings with varying TiO_2_ content levels. The obtained spectral data are presented in Figure 12. To ensure the accuracy and reliability of the XPS results, the standard C1s peak (binding energy 284.8 eV) was used as a calibration reference before spectral interpretation, effectively eliminating interference from instrumental systematic errors and surface charge effects on binding energy measurements. Figure 12 displays the calibrated XPS core spectra of Al 2p, Co 2p, Cr 2p, Fe 2p, Ni 2p, Ti 2p, and C1s on each sample surface, providing fundamental data support for subsequent element chemical state analysis.

XPS analysis revealed that the nuclear-level spectra of the major metal elements (Al, Co, Cr, Fe, Ni, Ti) in the passivation films of AlCoCrFeNi HEA coatings with varying TiO_2_ content amounts all exhibited a typical double-peak splitting characteristic. The splitting peaks corresponded to the metallic state and specific oxidation states of each element, respectively, with the energy difference between the two peaks remaining constant for each element. Detailed spectral analyses of the elements are as follows. The Al 2p spectrum split into two characteristic peaks, Al 2p_1_/_2_ and Al 2p_3_/_2_, corresponding to the Al^3+^ oxidation state (primarily in the form of Al_2_O_3_) and the metallic state Al, respectively, with a splitting energy of 6.98 eV. The Co 2p spectrum split into Co 2p_1_/_2_ and Co 2p_3_/_2_ peaks, corresponding to the Co^2+^ oxidation state (e.g., CoO) and the metallic state Co, respectively, with a splitting energy of 14.11 eV. The Cr 2p spectrum exhibited a distinct double-peak structure, with the Cr 2p_1_/_2_ and Cr 2p_3_/_2_ peaks corresponding to the Cr^3+^ oxidation state (primarily in the form of Cr_2_O_3_) and the metallic state Cr, respectively, with a splitting energy of 10.55 eV. The XPS spectra of Fe and Ni displayed similar splitting patterns: The Fe 2p spectrum split into Fe 2p_1_/_2_ and Fe 2p_3_/_2_ peaks, corresponding to the Fe^2+^ oxidation state (e.g., FeO) and the metallic state Fe, respectively, with a splitting energy of 4.51 eV. The Ni 2p spectrum splits into the Ni 2p_1_/_2_ and Ni 2p_3_/_2_ peaks, corresponding to the oxidized state Ni^2+^ (e.g., NiO) and the metallic state Ni, respectively, with a splitting energy of 4.57 eV. As a characteristic element introduced by TiO_2_ content, the Ti 2p energy spectrum also exhibits a double-peak splitting feature. The Ti 2p_1_/_2_ and Ti 2p_3_/_2_ peaks correspond to the oxidized state Ti^4+^ (i.e., TiO_2_) and the metallic state Ti, respectively, with a splitting energy of 4.89 eV.

XPS analysis revealed that the passivation films on the surfaces of three AlCoCrFeNi high-entropy alloy (HEA) coatings with different TiO_2_ content levels primarily consist of metal oxide components, including Al, Co, Cr, Fe, Ni, and Ti. Figure 12 presents the peak fitting results of the core energy-level spectra for each element, while Figure 12 visually illustrates the relative content ratios of metal oxide components in the passivation films, providing quantitative data to support subsequent studies on the correlation between composition and corrosion resistance. Existing research has confirmed that the stability of HEA surface passivation films is inversely correlated with Al content: when Al content is excessively high, Al_2_O_3_ gradually replaces Cr_2_O_3_ as the dominant component of the passivation film. Data in Figure 11 show that in samples doped with 0.5 wt.% and 1.0 wt.% TiO_2_, the relative content of Al^3+^ (corresponding to Al_2_O_3_) significantly increased to 57.95% and 47.22%, respectively. This is one of the key factors contributing to the relatively poor corrosion resistance of these samples [49]. Conversely, as TiO_2_ content increases, the relative content of Fe^2+^ (corresponding to FeO) and Cr^3+^ (corresponding to Cr_2_O_3_) in the passivation films exhibits a significant upward trend. Previous studies have demonstrated that chromium (Cr) plays a dominant role in the formation of passivation films on CoCrFeNi-based HEA coatings. The weak binding characteristics of Cr-Cr bonds enable easier adsorption of O^2−^ from corrosive media, leading to the formation of a dense Cr_2_O_3_ oxide film. This film layer effectively shields against corrosion from corrosive media due to its superior physical barrier properties and chemical stability [50,51]. Additionally, as the TiO_2_ content concentration increases, the relative content of Ti^4+^ (corresponding to TiO_2_) in the passivation film rises accordingly. Studies demonstrate that the enrichment of TiO_2_ in the passivation film significantly enhances the structural stability and corrosion resistance of the coating, thereby strengthening its protective effect on the substrate [52,53].

**Figure 13:**
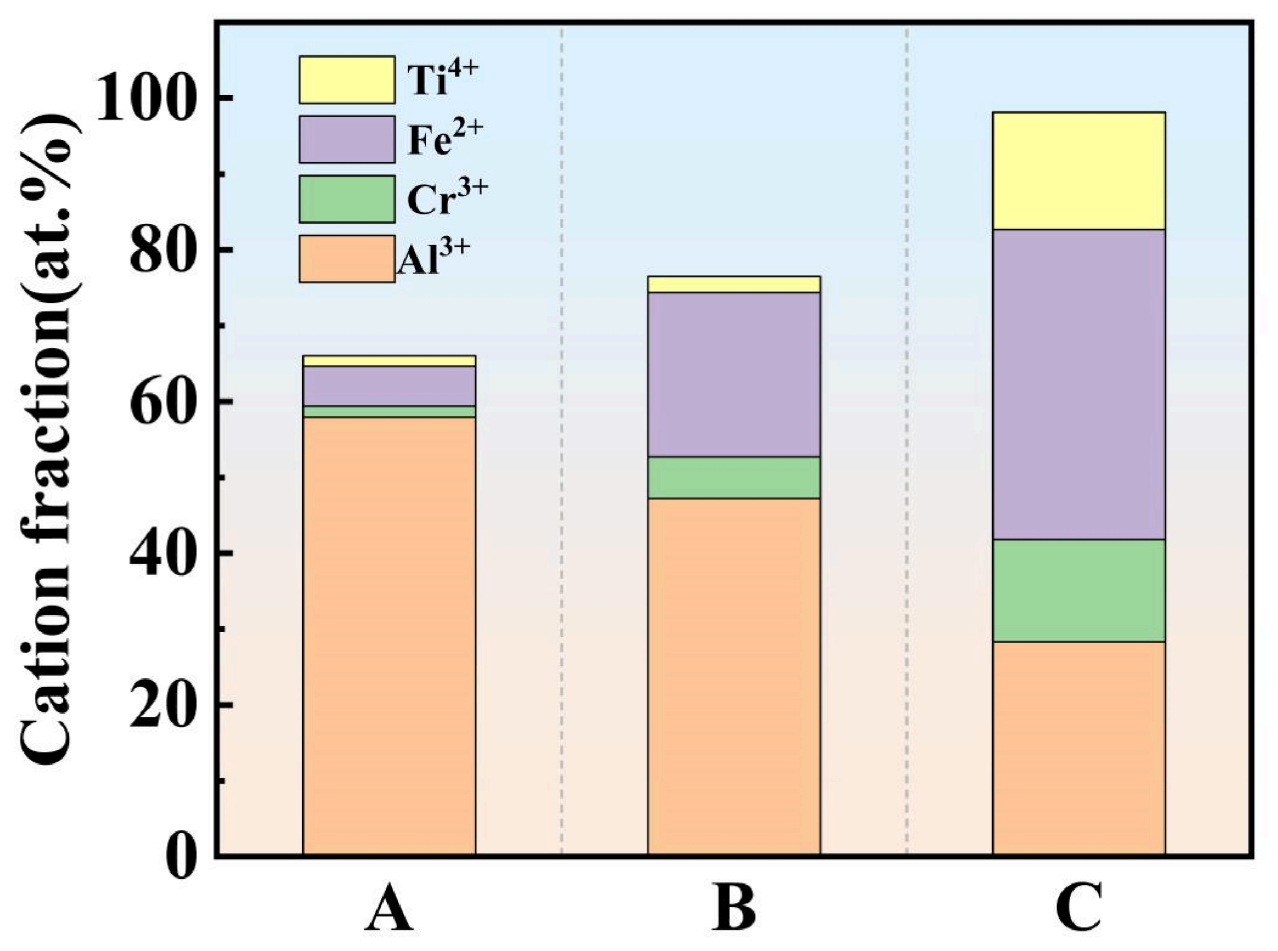
Relative content of cationic elements in the passivation film of AlCoCrFeNi HEA coatings with different TiO_2_ content: A. 0.5 wt.%; B. 1 wt.%; C. 1.5 wt.%.

A comprehensive analysis of the XPS results in Figure 13 and the composition ratio of oxide components in the passivation film reveals that with increasing TiO_2_ content, the oxide content of Fe^2+^ (corresponding to FeO), Cr^3+^ (corresponding to Cr_2_O_3_), and Ti^4+^ (corresponding to TiO_2_) in the HEA coating surface passivation film shows a continuous upward trend, while the oxide content of Al^3+^ (corresponding to Al_2_O_3_) decreases markedly. This compositional evolution enables AlCoCrFeNi HEA coatings with higher TiO_2_ content concentrations to form a denser passivation film, thereby substantially improving their corrosion resistance.

**Figure 14:**
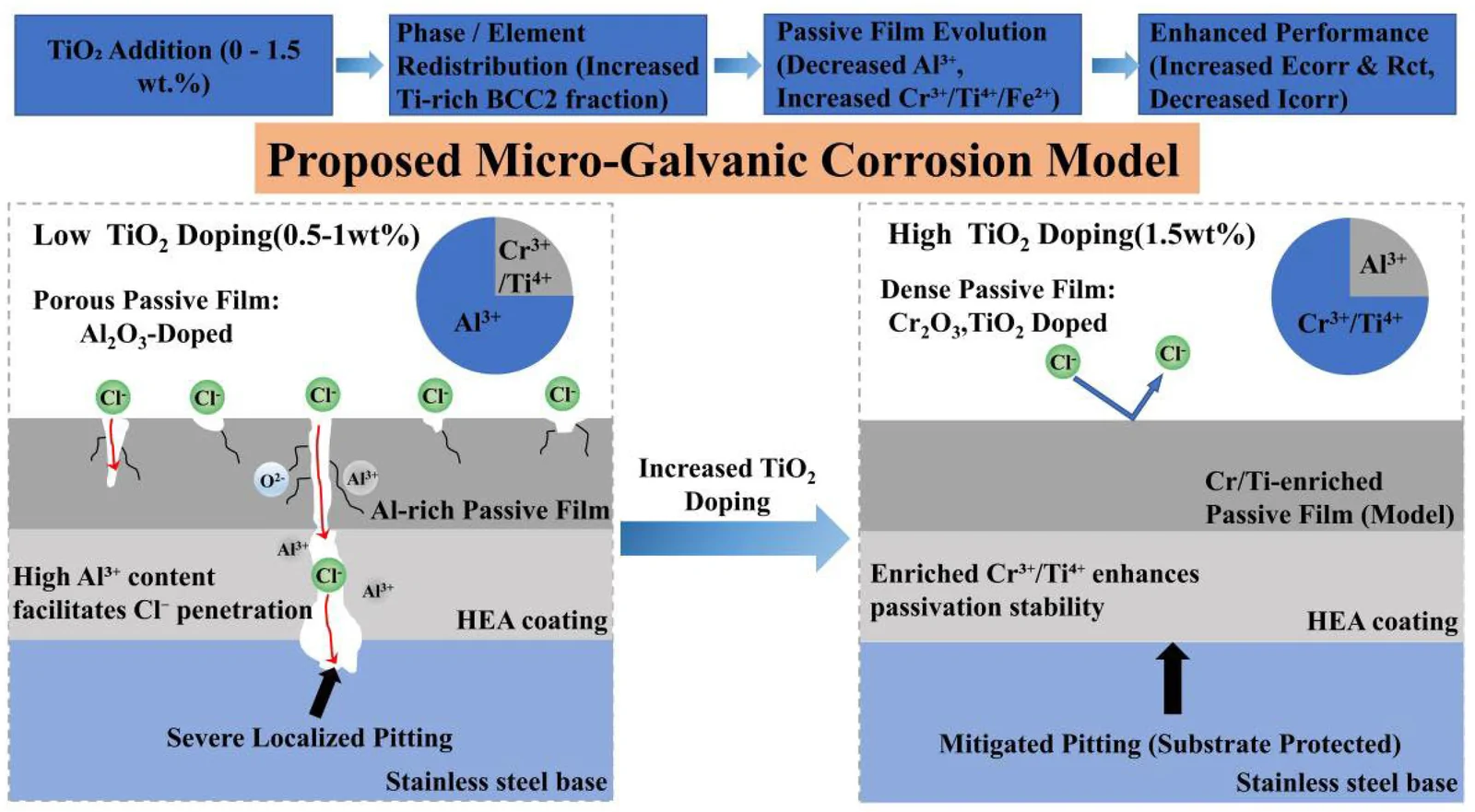
Proposed mechanistic model correlating TiO_2_ addition to the altered microgalvanic corrosion behavior of AlCoCrFeNi HEA coatings in a 3.5 wt.% NaCl solution.

Based on the preceding discussions, Figure 14 illustrates the corrosion mechanism of AlCoCrFeNi HEA in a 3.5 wt.% sodium chloride solution. For AlCoCrFeNi HEA coatings with low TiO_2_ content, chloride ions initially accumulate on the oxides and hydroxides formed on the alloy surface. Due to weak grain boundary stability, chloride ion adsorption causes rupture of the passivation film, exposing the substrate to the Cl^−^ solution and forming multiple small pits [54]. As corrosion progresses, the intact passivation film together with locally damaged regions establishes a large cathode–small anode structure, accelerating the formation and development of pitting nuclei, which in most cases further expand to form corrosion pores [55].

Based on the quantitative experimental evidence obtained in this study, increasing TiO_2_ addition demonstrably leads to a higher volume fraction of Ti–Cr-rich BCC2 regions and a fundamental shift in the passive film chemistry—specifically, increased relative content of Cr^3+^, Fe^2+^, and Ti^4+^ oxides at the expense of Al^3+^ oxides. These experimentally confirmed microstructural and chemical evolutions directly correlate with the significantly reduced pit depth and diameter observed macroscopically.

From these robust experimental observations, we mechanistically infer a microgalvanic corrosion model to explain the altered localized corrosion behavior. In this proposed model, the corrosion process is suggested to proceed through three distinct stages, as follows.

The HEA coating with higher TiO_2_ content exhibits a more corrosion-resistant BCC2 phase, leading to altered corrosion behavior that proceeds through three distinct stages. In the initial passivation stage (microscopic heterogeneity), a complete biphasic passivation film spontaneously forms on the coating surface, though the macroscopically continuous film demonstrates significant compositional and structural heterogeneity at the microscopic level. In the titanium-deficient BCC1-phase region, the passivation film contains numerous oxygen vacancies, grain boundary defects, and ion transport channels, resulting in poor film density and weak adhesion. Conversely, in the titanium-rich BCC2-phase region, the passivation film is dense, intact, and free of obvious defects, exhibiting robust adhesion to the substrate and superior passivation stability.

Corrosion initiation stage (Cl^−^ selective erosion): In a 3.5 wt.% NaCl solution, Cl^−^ ions—with their small radius and strong penetration capability—prioritize adsorption on the surface of the BCC1 phase, which exhibits higher electrochemical activity. These ions rapidly penetrate through oxygen vacancies and physical defects in the passivation film, undergo complexation reactions with metal cations to form soluble metal chlorides. The anodic reaction equation is as follows:

(4) $$\mathrm{Al}-3e^{-}\to {\mathrm{Al}}^{3+}$$

The oxygen reduction reaction at the cathode occurs on the outer surface near the pore opening, with the reaction equation as follows:

(5) $$O_{2}+4H_{2}O+4e^{-}\to \;4{\mathrm{OH}}^{-}$$

Corrosion progression stage (selective localized dissolution): As corrosion progresses, the difference in corrosion resistance between the two phases results in a typical non-uniform localized corrosion morphology. The titanium-deficient BCC1 phase undergoes irreversible matrix dissolution under continuous anodic dissolution and intra-pore autocatalytic acidification, forming large, deeply developed corrosion pits. In contrast, the titanium-rich BCC2 phase, protected by a highly stable composite oxide film, effectively resists Cl^−^ erosion and microgalvanic corrosion, maintaining a smooth and dense surface. This ultimately manifests as a differentiated corrosion pattern characterized by “selective dissolution of BCC1 phase with complete preservation of BCC2 phase.”

The microscopic regulation mechanism of TiO_2_ content concentration: TiO_2_ content significantly modulates the phase composition ratio of the coating. As TiO_2_ content increases, the volume fraction of the highly corrosion-resistant titanium-rich BCC2 phase in the HEA coating rises, while the area of the more susceptible titanium-poor BCC1 phase correspondingly decreases. This optimized phase structure fundamentally reduces pitting nucleation sites, effectively inhibits the initiation and propagation of corrosion pits, and substantially enhances the coating’s overall corrosion resistance.

## 4. Conclusions

In summary, the original contribution of this study lies in elucidating the high-temperature metallurgical synergy between TiO_2_ and an AlCoCrFeNi matrix during laser cladding, a mechanism distinctly different from the physical barrier effects typically observed in traditional TiC or TiO_2_ particle-reinforced composites. While previous studies have primarily demonstrated that inert ceramic additions physically impede localized corrosion, our work explicitly establishes a novel composition–microstructure–passivation relationship. We experimentally demonstrate that reactive TiO_2_ doping actively modifies the alloy’s phase constitution (promoting Ti-rich BCC2 regions) and fundamentally alters the passive film chemistry (enriching Cr and Ti oxides). This interactive mechanism offers a novel metallurgical pathway for designing high-performance, chloride-resistant HEA composite coatings.

This study investigated the effect of TiO_2_ content on the microstructure and corrosion resistance of laser-cladded AlCoCrFeNi HEA coatings in 3.5 wt.% NaCl solution. The main conclusions are as follows.

1. The optimal laser-cladding parameters were determined by orthogonal experimental design and range analysis: laser power of 1500 W, scanning speed of 40 mm/s, and powder feed rate of 11.9 g/min.
2. TiO_2_ doping promoted the formation of a Ti–Cr-rich BCC2 phase and enhanced Ti segregation in the AlCoCrFeNi coating. This indicates that TiO_2_ effectively modified the solidification behavior, phase constitution, and elemental distribution of the coating.
3. Electrochemical results showed that increasing TiO_2_ content reduced the corrosion current density, shifted the corrosion potential positively, and increased the charge-transfer resistance, thereby improving the corrosion resistance of the coating.
4. XPS analysis indicated that TiO_2_ content increased the relative content of Fe, Cr, and Ti oxides in the passive film while reducing the Al oxide fraction. The resulting dense and stable composite passive film effectively inhibited corrosive ion penetration and enhanced the overall corrosion resistance of the coating.

## Figures and Tables

**Table 1:** Representative studies on Ti-bearing or ceramic-modified HEA coatings.

HEA System	Process	Modification	Environment	Main Corrosion Response
HEA coating	High-speed laser cladding	TiC	—	TiC particle size affected corrosion behavior
FeNiCoCr	Laser cladding	TiC	3.5 wt.% NaCl	TiC type affected localized corrosion behavior
Ti-modified HEA	Laser cladding	Ti	NaCl solution	Ti altered phase/passivation behavior
Oxide-modified HEA	Laser cladding	Oxide	NaCl solution	Oxide addition affected microstructure and corrosion
Present work	Laser cladding	0–1.5 wt.% TiO_2_	3.5 wt.% NaCl	TiO_2_ content correlated with phase evolution, pitting and passive-film chemistry

**Table 2:** Orthogonal experimental process parameter design.

	Process Parameters		
	Laser Power	Scanning Speed	Power Feeding Rate
	P	V	F
	W	mm/s	g/min
1	900	35	3.9
2	900	40	6.5
3	900	45	9.0
4	900	50	11.9
5	1200	35	6.5
6	1200	40	3.9
7	1200	45	11.9
8	1200	50	9.0
9	1500	35	9.0
10	1500	40	11.9
11	1500	45	3.9
12	1500	50	6.5
13	1800	35	11.9
14	1800	40	9.0
15	1800	45	6.5
16	1800	50	3.9

**Table 3:** Experimental data for cross sections of AlCoCrFeNi HEA coatings.

Serial Number	Weld Width	Pool Depth	Melting Height	Dilution Ratio	Shape Factor
	W/mm	h/mm	Tc/mm	η	λ
1	2.65	0.18	0.77	0.43	3.44
2	2.76	0.19	0.60	0.41	4.60
3	2.80	0.15	0.51	0.41	5.49
4	2.61	0.15	0.42	0.39	6.21
5	2.97	0.27	0.84	0.4	3.54
6	2.86	0.23	0.59	0.38	4.85
7	2.86	0.24	1.06	0.44	2.70
8	2.75	0.19	0.74	0.45	3.71
9	3.08	0.25	0.96	0.43	3.21
10	3.08	0.22	1.11	0.16	2.77
11	3.02	0.33	0.73	0.31	4.14
12	2.95	0.25	0.74	0.39	3.99
13	3.80	0.30	1.31	0.44	2.90
14	3.70	0.27	1.32	0.45	2.80
15	3.60	0.25	1.01	0.43	3.56
16	3.56	0.42	0.86	0.34	4.14

**Table 4:** Range analysis in orthogonal experiments.

Sample Number	Laser Power/(w)	Scanning Speed/(mm/s)	Power Feeding Rate/(g/min)	Dilution Ratio (%)
1	1	1	1	0.43
2	1	2	2	0.41
3	1	3	3	0.41
4	1	4	4	0.39
5	2	1	2	0.4
6	2	2	1	0.38
7	2	3	4	0.44
8	2	4	3	0.45
9	3	1	3	0.43
10	3	2	4	0.16
11	3	3	1	0.31
12	3	4	2	0.39
13	4	1	4	0.44
14	4	2	3	0.45
15	4	3	2	0.43
16	4	4	1	0.34
k_1_	0.41	0.425	0.365	
k_2_	0.4175	0.35	0.4075	
k_3_	0.3225	0.3975	0.435	
k_4_	0.415	0.3925	0.3575	
R	0.095	0.075	0.0775	

**Table 5:** Dynamic polarization parameters of AlCoCrFeNi HEA coatings with different TiO_2_ content in 3.5 wt.% NaCl solution: A. 0 wt.%; B. 0.5 wt.%; C. 1 wt.%; D. 1.5 wt.%.

Doping Ratio	E_corr_ (V vs. SCE)	I_corr_ (A/cm^2^)
Sample A	−1.4185	2.3279 × 10^−4^
Sample B	−1.1768	1.4462 × 10^−5^
Sample C	−0.9466	7.5715 × 10^−6^
Sample D	−0.6841	2.2834 × 10^−6^

**Table 6:** Electrochemical impedance spectroscopy (EIS) parameters of AlCoCrFeNi HEA coatings with different TiO_2_ content in 3.5 wt.% NaCl solution.

Sample	Rs (Ω∙cm^2^)	Rct (Ω∙cm^2^)	Yo	*n*	χ^2^
0.5 wt.% TiO_2_	59.65 ± 2.15	1802.57 ± 85.30	2.68 × 10^−8^	0.78746	1.5 × 10^−4^
1 wt.% TiO_2_	59.06 ± 1.84	3347.01 ± 112.45	2.76 × 10^−8^	0.82151	1.8 × 10^−4^
1.5 wt.% TiO_2_	57.39 ± 2.01	4821.00 ± 145.20	3.12 × 10^−8^	0.96991	1.4 × 10^−4^

## Data Availability

The data presented in this study are not available on request from the corresponding author.
